# Does breastfeeding protect children from COVID-19? An observational study from pediatric services in Majorca, Spain

**DOI:** 10.1186/s13006-021-00430-z

**Published:** 2021-10-18

**Authors:** Sergio Verd, Jan Ramakers, Isabel Vinuela, Maria-Isabel Martin-Delgado, Aina Prohens, Ruth Díez

**Affiliations:** 1 Pediatric Unit. La Vileta Surgery. Department of Primary Care, Matamusinos Street, 07013 Palma de Mallorca, Spain; 2 Balearic Islands Health Research Institute (IdISBa), 79 Valldemossa Road, 07120 Palma de Mallorca, Spain; 3 Department of Pediatrics, Son Espases University Hospital, 79 Valldemossa Road, 07120 Palma de Mallorca, Spain; 4 Pediatric Unit. Santa Ponsa Surgery. Department of Primary Care, Riu Sil Street, 07180 Calvia, Mallorca Spain; 5 Department of Pediatrics, Son Llatzer University Hospital, Manacor Road, 07120 07198 Palma de Mallorca, Spain

**Keywords:** Child, Breastfeeding, COVID-19, Disease transmission, Oxidative stress, Immune responses, Symptoms

## Abstract

**Background:**

It has been demonstrated that children who had been breastfed remain better protected against various infections, and notably respiratory tract infections, well beyond infancy. Since the role of breastfeeding to explain why children are less affected by COVID-19 has not been studied until now, the aim of this study was to determine whether any history of breastfeeding reduces the incidence rate of COVID-19 in children.

**Methods:**

This was a secondary analysis of an observational study on clinical and epidemiological characteristics of pediatric COVID-19 in Majorca. A total of 691 children were recruited during the 5 months of August–December 2020. Eligible participants were children under 14 who were tested for SARS-CoV-2 in pediatric emergency services. The independent explanatory variable was any breastfeeding. Bivariate analyses were conducted through the Chi-square test, the Fisher’s Exact test or the Student’s T test.

All children had the same demographic, epidemiological and clinical data collected through a study team member interview and via the participants medical records.

**Results:**

Within the sample of children who visited emergency services with symptoms of potential COVID-19, we found higher prevalence of positive SARS-CoV-2 RT-PCR test results among those who were exclusively formula fed compared with those who were ever breastfed (OR 2.48; 95% CI 1.45, 3.51; *P* = 0.036).

**Conclusions:**

The present study suggests that ever breastfeeding reduces the risk of COVID-19 among children, as documented for other infections.

## Background

In contrast to other respiratory viruses, children have been less affected by COVID-19 than adults. Furthermore, for reasons that remain elusive, the vast majority of reported infections in children are mild or asymptomatic [1, 2]. Recent reviews have proposed a wide range of mechanisms for this difference, including factors such as concurrent infections, differences in microbiota, anti-oxidative properties, or protective effects of global active viral immunization of children from birth till 6 years [3].

However, as far as we know, the role of pre-exposure to mother’s milk as a protective factor against COVID-19 has not been critically reviewed. Human milk contains an array of antimicrobial, anti-inflammatory and immunomodulatory compounds that confer long-term benefits against infections years after lactation is terminated [4]. Since it is acknowledged that severe COVID-19 is associated with uncontrolled inflammation, and more recently research has focused on host iron dysregulation [5], breastfeeding may have a protective role to play. It has been reported worldwide that children who were ever breastfed show lower blood levels of both ferritin [6] and biomarkers that are severely increased during the cytokine burst (i.e., serum monocyte chemoattractant protein-1 or uric acid) than their formula fed peers [7–9]. Theoretically, these characteristics might counterbalance the deranged biochemical pathways leading to multi-organ failure in COVID-19.

Given this latter possibility and that a detailed examination of risk factors for COVID-19 in this unique population is needed, we used a cohort of children to explore the hypothesis that the risk of pediatric COVID-19 is attenuated among children who were ever breastfed. The secondary aim of this study was to compare the clinical spectrum of children attending emergency services (ESs) during the COVID-19 pandemic according to infant feeding categories. We expect to provide new insight into the emergence and early outcomes of children infected with COVID-19.

## Methods

This is a secondary analysis of COVID IB4221/20PI, an ongoing multicentric observational study of clinical and epidemiological features of children who present for care in one of thirteen participating pediatric ESs located in Majorca, Spain, during the late summer and autumn 2020 period of the COVID-19 pandemic.

### Study strategy

This study was conducted in two University Hospitals, a District Hospital, nine healthcare centers for community Pediatrics, and a Private Hospital, where COVID-19 testing was conducted free of charge.

### Study design and participants

The first aim of our study was to analyze the effect of exclusive formula feeding on the risk of testing positive for SARS-CoV-2. The secondary aim was to examine the relationship between breastfeeding type and symptoms or diagnosis of children attending ESs during the COVID-19 pandemic. Eligible participants were those younger than 14 years of age who presented to a participating ES for care and who were tested for SARS-CoV-2 because of suspected COVID-19, ambulatory care or scheduled hospital procedures on the 2nd and 16th of each month from August to December 2020. The SARS-CoV-2 test from nasopharyngeal swab specimens targeted RNA-dependent RNA polymerase (RdRP), Nucleocapsid (N), and Envelope (E) gene sequences.

### Data collection

At enrollment in the ES, regardless of SARS-CoV-2 test results, all children had the same information collected, including demographic, epidemiological and clinical data. A study team member completed the enrollment form using the participants medical records and through a caregiver interview. Data collected included calendar date and site of enrollment, recent travel, exposures, household conditions, age, sex, health information (e.g., infant feeding, past medical history, weight, height), onset of current illness (e.g., timing, symptoms), and laboratory investigations performed in the emergency services. Study team members asked specifically about fever, asthenia, pain, diarrhea or respiratory symptoms, as well as other new or worsening health problems, and recorded the discharge diagnoses. Any diagnoses of respiratory tract infection, including acute otitis media (AOM), was validated with clinical examination. Since heart conditions, cerebral palsy, and chronic diseases are associated with more severe COVID-19 and multi-organ involvement [10], past medical history has focused specially on these pre-existing comorbidities.

#### Growth patterns

Percentiles of body mass index (BMI) were determined according to the Spanish Orbegozo Foundation growth charts [11]. A child’s weight status was established as defined by the Centers of Disease Control [12]: individuals who had BMI values below the 5th percentile were classified as underweight, healthy weight was defined as a BMI at or above the 5th percentile and below the 85th percentile, overweight was defined as a BMI at or above the 85th percentile and below the 95th percentile, and obesity was defined as a BMI at or above the 95th percentile.

#### Estimation of breastfeeding rate

The independent explanatory variable was any breastfeeding. A brief interviewer-administered question was used to collect quality data recalled about lactation: “Was [child] ever breastfed directly at the breast?” Answer options: Yes / No / Don’t know / Refused. This is the first modified question of the validated Brief Breastfeeding and Milk Expression Recall Survey [13].

### Sample size

At the time of study development, the wide variation of COVID-19 incidence rate in pediatric patients (1 -16%) [14] precluded a robust sample size estimate. Hence, we have opted for a convenience sample of children screened for COVID-19 at the ES of Majorca during 5 months of August–December 2020, of the COVID-19 pandemic.

### Data analysis

Statistical analyses were performed with IBM SPSS v.23 statistical software. Categorical data were presented as percentages. Normally distributed variables were described by means ± standard deviations. Proportions were compared using the Pearson’s chi-squared or the Fisher’s exact tests. Differences between means were studied through the Student’s T test. A *P* value < 0.05 was considered statistically significant.

### Ethics

The study was approved by the Balearic Conjoint Health Research Ethics Review Board. There is no direct benefit of study participation. Weekly interim analysis allowed for real-time data sharing with regional policy makers. A research team member at each institution contacted in-person the guardian/caregiver/child to obtain written informed consent and assent, as appropriate.

Preregistration: Aspredicted Trials Registry number is #62721.

## Results

This sample consisted of 691 children with consistent information regarding infant feeding. The median age of breastfed children was 54.6 months (interquartile range Q_3_-Q_1_ = 77–27) and the median age of formula fed children was 48 months (Q_3_-Q_1_ = 87.75–17). Most children had no known comorbidities (81%), and they were predominantly male (55%). Sixty-eight per cent of participant children were ever breastfed, and 32 % were never breastfed. There were no differences between the groups in terms of age, sex, comorbidity, height or weight. However, there were some differences among breastfed and formula fed groups in terms of BMI and household. Lower BMI of children was positively linked to breastfeeding rates. In addition, breastfed children were found to live in households with fewer children, as opposed to formula fed children. Table 1 shows the mean values and standard deviations of the baseline characteristics of the study sample compared by feeding categories.

**Table 1:** Baseline and demographic characteristics of patients from thirteen participating pediatric Emergency Services in Majorca, Spain, between August and December 2020

Variables	No breastfeeding	Any breastfeeding	***P*** value
Number (%)	220 (32%)	471 (68%)	
Children’s characteristics			
Age (months)	67.2 ± 51.7	69.3 ± 54.3	0.611
Female/male gender	97/123	211/260	0.862
Height (cm)	120.9 ± 28.9	116.8 ± 33.9	0.515
Weight (kg)	24.0 ± 15.9	23.6 ± 16.1	0.762
Body mass index^a^ (kg/m^2^)			
underweight	0 (0%)	8 (8.3%)	
healthy weight	24 (65%)	50 (52%)	
overweight	5 (13.5%)	27 (28%)	
obese	8 (21.5%)	11 (11.7%)	0.042*
Known comorbidities	46 (20.4%)	88 (18.7%)	0.535
Household			
Number of bedrooms in the house	2.75 ± 0.74	2.62 ± 0.75	0.032*
Children under 5 in the household	0.78 ± 0.72	0.80 ± 0.69	0.742
Children under 10 in the household	1.30 ± 0.95	0.97 ± 0.84	< 0.0001***
Children under 15 in the household	2.25 ± 1.53	1.61 ± 1.31	< 0.0001***
Healthcare facility			
Private pediatric settings	77 (38.5%)	139 (30.1%)	
Public pediatric settings	123 (61.5%)	322 (69.9%)	0.011*
Urban pediatric settings	186 (84.5%)	384 (81.5%)	
Rural pediatric settings	34 (14.5%)	87 (18.5%)	0.331

Values are presented as number (%) or means ± standard deviations

^a^according to the Centers of Disease Control percentiles

* ≤ 0.05; *** < 0.0001

### Clinical outcomes

Our study showed that exclusive formula feeding was associated with a significant increase in the risk of headache (OR 4.40; 95% CI 1.31–14.79; *P* = 0.016), AOM (OR 2.04; 95% CI 1.08–3.82; *P* = 0.026), and positive SARS-CoV-2 RT-PCR test results in symptomatic children (OR 2.48; 95% CI 1.45–3.51; *P* = 0.036). The proportion of all exclusively formula fed children (with or without symptoms) who tested positive for SARS-CoV-2 was also higher compared with the group of ever breastfed children (with or without symptoms); this difference did not reach statistical significance (OR 2.18; 95% CI 1.19–3.17; *P* = 0.056) but may be clinically significant. Only one patient who tested positive for SARS-CoV-2 was admitted into hospital; he was a never breastfed 13 years old boy. There were no significant differences between the two groups for the rest of observed clinical outcomes (Table 2).

**Table 2:** Identified medical problems by feeding type from thirteen participating pediatric Emergency Services in Majorca, Spain, between August and December 2020

Clinical or laboratory features	No breastfeeding	Any breastfeeding	***P*** value	OR (95%CI)
Number (%)	220 (32%)	471 (68%)		
Presenting Symptoms				
Fever	86 (39.1%)	184 (39.1%)	0.995	1.00 (0.72, 1.38)
Asthenia	20 (9.1%)	48 (10.2%)	0.651	0.88 (0.50, 1.52)
Dyspnea	9 (4.1%)	33 (7.0%)	0.069	0.50 (0.23, 1.05)
Coughing	64 (29.1%)	133 (28.2%)	0.817	1.04 (0.73, 1.48)
Sore throat	30 (13.6%)	70 (14.9%)	0.669	0.90 (0.57, 1.43)
Ear ache	14 (6.4%)	19 (4.0%)	0.184	1.61 (0.79, 3.28)
Abdominal pain	28 (12.7%)	48 (10.2%)	0.321	1.28 (0.78, 2.11)
Diarrhoea	25 (11.4%)	40 (8.5%)	0.230	1.38 (0.81, 2.34)
Diagnosis				
Acute pharyngitis	9 (4.1%)	20 (4.2%)	0.924	0.96 (0.43, 2.14)
Acute otitis media	20 (9.1%)	22 (4.7%)	0.026*	2.04 (1.08, 3.82)
Laryngitis	3 (1.4%)	7 (1.5%)	0.900	0.91 (0.23, 3.57)
Asthma	8 (3.6%)	27 (5.7%)	0.245	0.62 (0.27, 1.38)
Acute gastroenteritis	20 (9.1%)	31 (6.6%)	0.241	1.41 (0.78, 2.55)
Acute appendicitis	0 (0%)	2 (0.4%)		
Seizures	1 (0.5%)	4 (0.8%)	0.332	0.23 (0.01, 4.39)
Headache	8 (3.6%)	4 (0.8%)	0.016*	4.40 (1.31, 14.79)
Febrile syndrome	22 (10%)	37 (7.9%)	0.348	1.30 (0.74, 2.26)
Fracture	1 (0.5%)	7 (1.5%)	0.265	0.302 (0.03, 2.47)
Dermatitis	4 (1.8%)	13 (2.8%)	0.459	0.65 (0.21, 2.02)
Rash	2 (0.9%)	6 (1.3%)	0.677	0.71 (0.14, 3.55)
Symptomatic patients	203 (92.3%)	431 (91.5%)	0.73	0.90 (049, 1.63)
Positive results of SARS-CoV-2 RT-PCR tests				
From symptomatic and asymptomatic patients	8 (3.6%)	8 (1.6%)	0.056	2.18 (1.19, 3.17)
From symptomatic patients	8 (3.9%)	7 (1.6%)	0.036*	2.48 (1.45, 3.51)

*Abbreviations:* * ≤ 0.05; CI Confidence interval, OR Odds ratio, RT-PCR Real-time polymerase chain reaction, SARS-CoV-2 Severe acute respiratory syndrome coronavirus 2

## Discussion

We confirm that the risk of any COVID-19, AOM, or headache in children is amplified for those who were never breastfed. In fact, approximately 1 in 25 had tested positive for SARS-CoV-2; on the other hand, among ever breastfed children, 1 in 60 had tested positive for SARS-CoV-2.

We have not identified previous studies that have examined whether breastfeeding affords long-term protection from SARS-CoV-2. The risk of COVID-19 presumably reflects a balance between the rate of SARS-CoV-2 transmission to close contacts, and the individual protective immune response to respiratory viruses.

Another important question is whether breastfeeding from the start could modulate the immune response to fight SARS-CoV-2 a few years later. There is good evidence for enhanced protection against infections years after lactation is terminated [4]. A systematic review has concluded that 13 out of 16 studies show that breastfeeding protects infants against respiratory tract infections [15]. Children who had been breastfed remain better protected against respiratory tract infections for 7 years compared with those not breastfed. Breastfeeding predicts lower incidence of wheezing bronchitis for up to 6 years of age [16], and of meningitis for up to 10 years later, whereas no relation seems to exist after 15 years and beyond [17]. Early consumption of mother’s milk also confers long-term benefits against acute appendicitis [18] and tonsillectomy [19]. The mechanisms through which breastfeeding could have a long-lasting impact on infectious disease are multiple [3, 4] including interfering with the oxidative stress, which is a key player in severe COVID-19. Oxidative stress is a characteristic of the obese state, and it is to note that the prevalence of obesity among never breastfed is approximately twice as much as among ever breastfed children of our cohort (21.5% versus 11.7%).

Concerning ear infections, a 2015 meta-analysis [20] found consistent evidence of a protective effect of breastfeeding on AOM occurrence. In general, there was an effect of breastfeeding on the risk of AOM during the first 2 years of life, but an effect lasting for 6 years was also found.

The protective effect of breastfeeding on the development of headache remains controversial. Breastfeeding was found associated with reduced recurrent primary headache prevalence in a case-control study in which 61% of cases versus 95% of controls had been breastfed (*p* < 0.0005) [21]. Our results agree with this report despite the fact that we focus on acute headache instead of recurrent headache. However, a subsequent study failed to find a link between breastfeeding duration and headache development [22]. To our knowledge, there is no further research on this association.

With regards to the household characteristics, Woods et al. have recently reported that living with children was associated with an attenuated risk of any COVID-19 among adults in the household [23]. Given that we report that households with never breastfed children were more likely to include more children under 15, the higher rate of COVID-19 among never breastfed children in our sample is not likely to be associated to their specific indoor exposures.

To sum up, our current study reports for the first time that ever breastfed children remained at lower risk of COVID-19 during August–December 2020 than formula fed children. This adds to a large body of evidence that has been accumulating for three decades to suggest a strong (negative) correlation between breastfeeding and the incidence of infection 5 to 10 years later.

### Limitations

This is an observational study of less than one thousand children, our results may not be applicable to different populations. Our sample covers a wide age range (0–14 years), while previous research associates breastfeeding to lower risk of infection up to no more than 10 years beyond the lactation period. The wide variation of pediatric COVID-19 incidence does not allow for optimal sample size calculation, and the low number of positive SARS-CoV-2 RT-PCR results preclude a multivariate analysis of our data. There are several possible explanations for any patterns found. This study cannot establish whether any link is due to direct effects (breastfeeding) or indirect effects (environmental exposures of breastfed children). In addition, we have no data about the mitigation measures in the childcare/educational settings they attended (e.g. masking, and smaller, cohort groups). These variables may be relevant to get a sense of risk of exposure to SARS-CoV-2. Finally, it is also to be noted that the effects of breastfeeding are dose-dependent, hence we must acknowledge that “ever breastfeeding” is a generally problematic variable.

## Conclusions

This study has raised a new issue about COVID-19 in children. By studying children who attended emergency services at a time of community transmission of COVID-19, we found that ever breastfeeding may protect children against infection. This short report supports that there may be prolonged protection against infections following breastfeeding.

## Data Availability

The datasets generated and/or analysed during the current study are not publicly available since patients could be identified by knowing their date of birth and date of attending the health service, but are available from the corresponding author on reasonable request.

## Abbreviations

AOM: Otitis media
BMI: Body mass index
ESs: Emergency services

## References

[1] Mantovani A, Rinaldi E, Zusi C, Beatrice G, Saccomani MD, Dalbeni A (2021). Coronavirus disease 2019 (COVID-19) in children and/or adolescents: a meta-analysis. Pediatr Res.

[2] Yasuhara J, Kuno T, Takagi H, Sumitomo N (2020). Clinical characteristics of COVID-19 in children: a systematic review. Pediatr Pulmonol.

[3] Zimmermann P, Curtis N (2020). Why is COVID-19 less severe in children? A review of the proposed mechanisms underlying the age-related difference in severity of SARS-CoV-2 infections. Arch Dis Child.

[4] Li R, Dee D, Li CM, Hoffman HJ, Grummer-Strawn LM (2014). Breastfeeding and risk of infections at 6 years. Pediatrics.

[5] Cavezzi A, Troiani E, Corrao S (2020). COVID-19: hemoglobin, iron, and hypoxia beyond inflammation. A narrative review. Clin Pract.

[6] Engle-Stone R, Aaron GJ, Huang J, Wirth JP, Namaste SM, Williams AM, Peerson JM, Rohner F, Varadhan R, Addo OY, Temple V, Rayco-Solon P, Macdonald B, Suchdev PS (2017). Predictors of anemia in preschool children: biomarkers reflecting inflammation and nutritional determinants of anemia (BRINDA) project. Am J Clin Nutr.

[7] Wasilewska A, Tenderenda E, Taranta-Janusz K, Tobolczyk J, Stypułkowska J (2012). Markers of systemic inflammation in children with hyperuricemia. Acta Paediatr.

[8] Roszkowska R, Taranta-Janusz K, Tenderenda-Banasiuk E, Wasilewska A (2014). Increased circulating inflammatory markers may indicate that formula-fed children are at risk of atherosclerosis. Acta Paediatr.

[9] Verd S, Fraga G (2012). Markers of low-grade inflammation, breastfeeding and uric acid concentration. Acta Paediatr.

[10] Harman K, Verma A, Cook J, Radia T, Zuckerman M, Deep A, Dhawan A, Gupta A (2020). Ethnicity and COVID-19 in children with comorbidities. Lancet Child Adolesc Health.

[11] Sobradillo B, Aguirre A, Aresti U (2004). Curvas y tablas de crecimiento. Estudios longitudinal y transversal. [Growth standards. Longitudinal and transversal surveys].

[12] Centers for Disease Control and Prevention (2018). Overweight & Obesity. Defining childhood obesity.

[13] Keim SA, Smith K, Ingol T, Li R, Boone KM, Oza-Frank R (2019). Improved estimation of breastfeeding rates using a novel breastfeeding and milk expression survey. Breastfeed Med.

[14] Han X, Li X, Xiao Y, Yang R, Wang Y, Wei X (2021). Distinct characteristics of COVID-19 infection in children. Front Pediatr.

[15] Duijts L, Ramadhani MK, Moll HA (2009). Breastfeeding protects against infectious diseases during infancy in industrialized countries. A systematic review. Matern Child Nutr.

[16] Wilson AC, Forsyth JS, Greene SA, Irvine L, Hau C, Howie PW (1998). Relation of infant diet to childhood health: seven year follow up of cohort of children in Dundee infant feeding study. BMJ.

[17] Silfverdal SA, Bodin L, Olcén P (1999). Protective effect of breastfeeding: an ecologic study of Haemophilus influenzae meningitis and breastfeeding in a Swedish population. Int J Epidemiol.

[18] Pisacane A, de Luca U, Impagliazzo N, Russo M, de Caprio C, Caracciolo G (1995). Breast feeding and acute appendicitis. BMJ.

[19] Pisacane A, Impagliazzo N, De Caprio C, Criscuolo L, Inglese A, MCM PDS (1996). Breast feeding and tonsillectomy. BMJ.

[20] Bowatte G, Tham R, Allen K, Tan D, Lau M, Dai X, Lodge CJ (2015). Breastfeeding and childhood acute otitis media: a systematic review and meta-analysis. Acta Paediatr.

[21] Pogliani L, Spiri D, Duca PG, Dilillo D, Agostoni C, Penagini F (2008). Breastfeeding and headache, is there a protective effect?. Arch Dis Child.

[22] Pogliani L, Spiri D, Penagini F, Di Nello F, Duca P, Zuccotti GV (2011). Headache in children and adolescents aged 6-18 years in northern Italy: prevalence and risk factors. Eur J Paediatr Neurol.

[23] Wood R, Thomson E, Galbraith R, Gribben C, Caldwell D, Bishop J, et al. Sharing a household with children and risk of COVID-19: a study of over 300 000 adults living in healthcare worker households in Scotland. Arch Dis Child. 2021. 10.1136/archdischild-2021-321604 Epub ahead of print. 10.1136/archdischild-2021-321604 PMC7985971 33737319

